# Implications of life history uncertainty when evaluating status in the Northwest Atlantic population of white shark (*Carcharodon carcharias*)

**DOI:** 10.1002/ece3.6252

**Published:** 2020-04-28

**Authors:** Heather D. Bowlby, A. Jamie F. Gibson

**Affiliations:** ^1^ Population Ecology Division Science Branch, Fisheries and Oceans Canada Dartmouth NS Canada

**Keywords:** endangered species conservation, exploitation risk, fishery removals, life history analyses, white shark

## Abstract

To effectively protect at‐risk sharks, resource managers and conservation practitioners must have a good understanding of how fisheries removals contribute to changes in abundance and how regulatory restrictions may impact a population trajectory. This means they need to know the number of animals being removed from a population and whether a given number of removals will lead to population increases or declines. For white shark (*Carcharodon carcharias*), theoretical quantities like the intrinsic rate of population increase or rebound potential (ability to increase in size following decline) are difficult to conceptualize in terms of real‐world abundance changes, which limits our ability to answer practical management questions. To address this shortfall, we designed a simulation model to evaluate how our understanding of longevity and life history variability of white shark affects our understanding of population trends in the Northwest Atlantic. Then, we quantified the magnitude of removals that could have caused historical population declines, compared these to biologically based reference points, and explored the removal scenarios which would result in population increase. Our results suggest that removals on the order of 100s of juveniles per year could have resulted in population‐level declines in excess of 60% during the 1970s and 1980s. Conservation actions implemented since the 1990s would have needed to be nearly 100% effective at preventing fishing mortality in order for the population to double in abundance over the last 30 years. Total removals from all fleets needed to be exceptionally small to keep them below biological reference points for white shark in the Northwest Atlantic. The population's inherent vulnerability to fishing pressure reaffirms the need for restrictive national and international conservation measures, even under a situation of abundance increase.

## Graphical abstract

We designed a simulation model to evaluate how our understanding of longevity and life history variability of white shark affects our understanding of population trends in the North Atlantic. Then, we quantified the magnitude of removals that could have caused historical population declines, compared these to biologically based reference points, and explored the removal scenarios which would result in population increase. Our results demonstrate the inherent vulnerability of the species to fishing pressure and reaffirm the need for restrictive international conservation measures to protect the population, even under a situation of abundance increase.

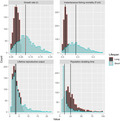

## 1 INTRODUCTION

The ecological consequences of human activities need to be identified and predicted in a context and at a scale relevant to resource managers (Fausch, Torgensen, Baxter, & Li, 2002). To effectively protect at‐risk sharks, managers must understand how exploitation contributes to changes in abundance, how life history characteristics influence population growth or the potential for recovery, and how regulatory restrictions may impact a population trajectory. Intuitively, these processes should be quantified relative to the size of the population (i.e., relative to an absolute number of animals) to make this information most useful (Driscoll & Lindenmayer, 2012). This is possible for data‐rich species, where landings, discards, and other data can be evaluated in an assessment model, which estimates a population's abundance, trajectory, and status relative to reference points. Predicting abundance or evaluating status is more problematic for data‐poor species in which incidental captures in fisheries are rare and sporadic (Punt, Smith, & Smith, 2011). White shark are a good example: For any given population, individuals are encountered infrequently over a vast geographical range (Bonfil et al., 2005; Skomal, Braun, Chisholm, & Thorrold, 2017), interact with diverse fisheries in national and international waters (Dewar et al., 2013; Queiroz et al., 2019), and thus have limited potential for using catches to monitor absolute (as opposed to relative) abundance (Baum et al., 2003; Burgess et al., 2005; Curtis et al., 2014). A reliable time‐series of total removals for white shark in the Northwest Atlantic is not available due to the need to identify and scale up observed interactions from multiple fleets where observation rates tend to be low (Curtis et al., 2018; Dulvy et al., 2008). Catch‐per‐unit‐effort (CPUE) indices of relative abundance coupled with life history information become the primary data sources that can be used to assess status.

Owing to several well‐known limitations of these data sources, uncertainties remain in our understanding of white shark life history as well as population trajectory in the Northwest Atlantic. Relative abundance indices are rarely proportional to actual abundance because of numerous factors affecting catch rates (reviewed in Maunder et al., 2006). Although a 79% decline from 1986 to 2000 in white shark abundance was estimated from commercial logbook data from the US Tuna/Swordfish fleet (Baum et al., 2003), their analysis focused on a single data set in exclusion of others, could have contained species identification issues, and was affected by a change in shark reporting requirements *circa* 1993 (Burgess et al., 2005). More recently, an analysis of several shark‐directed CPUE indices as well as sightings records suggested a similar overall rate of population decline (63%–73%), but one that occurred earlier, in the 1970s and 1980s (Curtis et al., 2014). Species identification issues as well as potential under‐ or over‐reporting of white shark may not be as prevalent in the data sources analyzed by Curtis et al. (2014), so this trend is expected to be more robust. Relative to life history data, targeted lethal sampling to evaluate biological characteristics is neither feasible nor desirable for populations that are currently at low abundance and have such low intrinsic rates of increase (Hammerschlag & Sulikowski, 2011; Heupel & Simpfendorfer, 2010). Information on maturation and reproduction in particular are limited for the Northwest Atlantic population of white shark, being based on extremely few samples or inferred from other populations and/or related species (Bruce, 2008; Dillingham et al., 2016). Additionally, the strongest validation method for age in elasmobranchs is bomb radiocarbon (Matta, Tribuzio, Ebert, Goldman, & Gburski, 2017), yet the behavioral characteristics of white shark (including wide‐ranging movements, sporadic feeding, and ontogenetic shifts in habitat use) complicate the choice of reference chronology and thus the validation of age (Andrews & Kerr, 2015; Cailliet, 2015). Recent research suggests that validated ages are approximately 30 years greater than those from previous unvalidated vertebral counts (Natanson & Skomal, 2015), and there is evidence that vertebral growth may slow in larger individuals leading to an underestimation of age (Andrews & Kerr, 2015; Hamady, Natanson, Skomal, & Thorrold, 2014).

From analyses of life history characteristics, the vulnerability of chondrichthyan fishes to exploitation is well established (Hutchings, Myers, García, Lucifora, & Kuparinen, 2012) and for white shark, has been used to rationalize some of the highest levels of global protection for any shark species (Curtis et al., 2014; Hillary et al., 2018). Relative to other fishes, their high juvenile survival, late age at maturity and long lifespans buffer the effects of short‐term environmental variability on population dynamics (Kindsvater, Mangel, Reynolds, & Dulvy, 2018), yet result in low intrinsic rates of population growth (*r*_max_ e.g., Gedamke, Hoenig, Musick, DuPaul, & Gruber, 2007; Cortés, 2016) or rebound potential (e.g., Au, Smith, & Show, 2015; Smith, Au, & Show, 1998) as well as slow population recovery rates following depletion (Hutchings et al., 2012). Metrics such as *r*_max_ or rebound potential can be difficult to conceptualize in terms of what specific values represent for real‐world changes in population size. For example, would removals of 100 animals per year cause population decline for a species with an *r*_max_ of 0.04? How much would the population trajectory change if removals were doubled? This limits our ability to answer practical management questions that arise, particularly those related to the magnitude of removals that may jeopardize population recovery. Ultimately, we are left with a good understanding of which shark species are the most vulnerable to exploitation, but little knowledge of what that vulnerability means in a practical context for a specific species.

To support conservation goals, our objective was to evaluate how greater longevity affects our understanding of population dynamics, relative abundance trends and vulnerability to exploitation for white shark in the Northwest Atlantic. In doing so, we were able to assess how effective conservation measures may have needed to be to match perceptions of recent population growth, as well as to quantify the possible magnitude of historical removals and compare them to reference points derived from the same life history. Given the need to determine current status without having to impact the current population, we used a simulation model to describe life history dynamics and evaluate abundance change, and incorporated both demographic and environmental variability to increase biological plausibility. By considering two specific life history scenarios, we demonstrated how increased longevity in white shark can affect the magnitude of removals the population can sustain. We also assessed the compatibility of different sources of life history information given our new understanding of longevity and identified pressing areas for research for this endangered iconic predator.

## 2 MATERIALS AND METHODS

There were two main components to our simulation: (a) a life table analysis used to estimate the capacity for population growth; and (b) population projections that modeled abundance in a particular year as a function of abundance in the previous year, given life history parameters and exploitation rates. We used Monte‐Carlo random sampling to incorporate uncertainty in the life table analysis and projections (Cortés, 2002; Dillingham et al., 2016; Dulvy, Pardo, Simpfendorfer, & Carlson, 2014), similar to the methods developed by Caswell, Brault, Read, and Smith (1998) and Dans, Koen Alonso, Pedraza, and Crespo (2003) for demographic analyses of cetaceans, and Campana, Gibson, Brazner, Marks, and Joyce (2008) for basking shark. For the population projections, we considered an exponential model to be appropriate given how severely the white shark population in the Northwest Atlantic was reduced in size (Curtis et al., 2014).

Aging techniques for sharks have substantially evolved in recent decades (Cailliet, 2015; Matta et al., 2017). For white shark, the application of modern age validation techniques has resulted in the possibility that the Northwest Atlantic population lives much longer than previously thought, where longevity could be in excess of 70 years (Hamady et al., 2014; Natanson & Skomal, 2015). We demonstrate how this shift in longevity would influence our understanding of population dynamics by developing life tables that represent short and long lifespans.

### 2.1 Estimating the capacity for population growth

Life table analyses are frequently used to determine the intrinsic rate of population growth (*r*) from demographic information (Cortés, 2016; Mollet & Cailliet, 2002). Such analyses are well suited for use in shark species given their well‐defined reproductive cycle and high rates of survival (Cortés, 1998). Calculations use estimates of survival rates and fecundity, as well as information on the timing of maturation as input parameters. From average life history rates, there are several methods for estimating *r* (Cortés, 2016) and we used a derivation of the Euler–Lotka equation (McAllister, Pikitch, & Babcock, 2001). This model is density independent, where the estimate of *r* is equivalent to the maximum rate of population growth (*r*_max_) in a density‐dependent model. It represents the maximum rate the population can increase from a severely depleted population size, provided the life history parameters used in the calculation represent a severely depleted population (Gedamke et al., 2007; McAllister et al., 2001). This is likely for white shark in the Northwest Atlantic, given the timing and severity of population declines (Curtis et al., 2014). We note that alternate metrics of population growth potential (i.e., other than *r* or *r*_max_) are less appropriate for these demographic analyses given the extent of population decline. The most well‐known alternative would be rebound potential, which describes the capacity for the population to increase from abundance at MSY (Au et al., 2015; Smith et al., 1998; Smith, Au, & Show, 2008).

The Euler–Lotka equation:

(1) $$1=\sum_{x=0}^{A}e^{-rx}m_{x}l_{x}$$

is a discrete approximation of an integral (McAllister et al., 2001), which means it does not have an analytical solution and *r* is estimated through numerical minimization. Here, *A* is the maximum age, $l_{x}$ is survival to age *x* ( $l_{0}=1$ ), and $m_{x}$ is the expected reproductive output at age *x*. Survival to each age is calculated as:

(2) $$l_{x}=\prod_{i=0}^{x-1}e^{-\left(M_{i}+F_{i}\right)},$$

where *M* is the instantaneous natural mortality rate and *F* is the instantaneous fishing mortality rate, the latter of which is set to zero when estimating intrinsic rates of population growth. Survivorship accounts for both juvenile and adult survival. We set up the model as a female‐only model relative to reproductive output, which is calculated from female age at maturity, the sex ratio at birth (here assumed to be 50:50), and fecundity of females. Where possible, we used values from the Northwest Atlantic population in the parameterization, recognizing that life history rates tend to be relatively uncertain for white shark in general (Bruce, 2008; Huveneers et al., 2018). Previous analyses of extinction risk (e.g., García, Lucifora, & Myers, 2008) and rebound potential (e.g., Smith et al., 1998) assumed an age of maturity of 9–10 for females when maximum ages were ~40, similar to our short lifespan scenario (Table 1). We based the age at maturity and longevity for the long lifespan scenario on Natanson and Skomal (2015), although we recognize that the maximum validated age for females was 40 as opposed to 70 for males. The length of the gestation period for white shark is thought to be around 20 months (Bruce, 2008; Christiansen et al., 2014). Postpartum females may require a year or more to recondition following pregnancy, leading to a reproductive cycle of 2–3 years (Dewar et al., 2013; Domeier & Nasby‐Lucas, 2013). Litter size has not been observed in the Northwest Atlantic, but may vary from 2 to 17 (Christiansen et al., 2014) and has been assumed to average in the range of 6–10 (50:50 sex ratio) in previous demographic analyses (e.g., Cortés, 2002; García et al., 2008; Smith et al., 1998). We used the same values for the length of the reproductive cycle as well as female litter size in the two longevity scenarios (Table 1).

**TABLE 1:** Life history parameter values used to calculate the potential for population increase (*r*) for the short and long lifespan scenarios

Lifespan	Parameter	Minimum	Deterministic value	Maximum
Short	Age at maturity	8	9.5	11
Short	Female litter size	2	4	6
Short	Gestation period	2	2.5	3
Short	Maximum age	35	40	45
Short	Natural mortality	0.062	0.112	0.162
Long	Age at maturity	25	30	35
Long	Female litter size	2	4	6
Long	Gestation period	2	2.5	3
Long	Maximum age	60	70	80
Long	Natural mortality	0.053	0.063	0.073

Note

The median was taken as the deterministic value and the minimum and maximum provide the range used in Monte‐Carlo sampling.

Calculating survivorship relies on having some estimate of the natural mortality rate (*M*) of the population. We explored several different methods to estimate natural mortality, including ones based on average lifespan (Pardo, Kindsvater, Reynolds, & Dulvy, 2016), and those based on maximum observed or assumed age (Kenchington, 2014). Our chosen estimator for *M* used the geometric mean regression equation for marine mammals originally done by Hoenig (1983). This regression technique was one of the few options that performed adequately for sharks in simulation analyses (Kenchington, 2014) and is suited to data that contain uncertainty in the estimate of maximum age. This estimator is commonly used in demographic analyses of sharks to approximate *M*, as either the only option (e.g., Au et al., 2015; Smith et al., 1998) or as one of multiple options (e.g., Cortés, 2002; García et al., 2008; Gedamke et al., 2007). For white shark specifically, both empirical estimates of natural mortality (0.079; Benson et al., 2018) and those derived through other modeling approaches (0.047–0.068; Cortés, 2016; Mollet & Cailliet, 2002) suggest *M* is similar to that calculated for the long lifespan scenario.

There are several ways that fishing mortality or survival rates may change over ontogeny, where differences in survival or susceptibility to fisheries among ages are expected (Benson et al., 2018; Dewar et al., 2013). The annual survival rates for the two longevity scenarios were 89% for the short lifespan and 94% for the long lifespan. Given similarities among the assumed maximum age, the value for the short lifespan falls within the range used in other age‐structured demographic analyses of white shark (0.81–0.92; Cortés, 2002; Dillingham et al., 2016). For white shark to live longer, mean survival should be higher, as in the long lifespan. For fishing mortality, differences in selectivity patterns are expected among the range of fisheries that would interact with white shark in the Northwest Atlantic (Curtis et al., 2014; Queiroz et al., 2019). Given the observation that fishing mortality is extremely low for adults but can be substantial for juveniles in the Northeast Pacific (Dewar et al., 2013), we assumed constant fishing mortality rates for juveniles and no fishing mortality on adults.

To allow for life history uncertainty within each life span scenario, we gave specific vital rates a lower and an upper boundary (minimum and maximum values) and used Monte‐Carlo (MC) sampling (e.g., Cortés, 2002; Dulvy et al., 2014) from assumed uniform distributions within these bounds to generate estimates of *r* (Table 1). We summarized the two longevity scenarios from their MC distributions (median and 80% percentiles) by four quantities: *r*, the lifetime reproductive rate (females per female), population doubling time under no fishing mortality (years), and the instantaneous fishing mortality rate that would prevent population increase ( $F_{\text{crit}}$ ). Lifetime reproductive rates represent the total number of adult female offspring produced by a single adult female during their lifetime, and $F_{\text{crit}}$ represents the fishing mortality rate at which the population growth rate is zero. To ensure variability between the two longevity scenarios was of a comparable magnitude, we set the bounds for age at maturity, natural mortality, and maximum age as similar percentages above and below the median value.

### 2.2 Using population decline rates to evaluate historical removals

The most recent estimates of population trajectory come from a hierarchical analysis by Curtis et al. (2014), who reported declines of 63%–73% during the 1970s and 1980s. This suggests that population size in the 1990s would have been very small, particularly given that white shark are naturally less abundant than species at a lower trophic levels (Burgess et al., 2014; Huveneers et al., 2018). Based on abundance estimates for specific aggregations (e.g., Burgess et al., 2014; Hillary et al., 2018), we expect that 2,000–3,000 animals is a realistic magnitude for population size in the Northwest Atlantic following the declines and prior to rebuilding, although actual abundance has not been determined at any time for this population. In the simulation model, we projected the population backwards from this assumed size to evaluate how many individuals may have been removed to cause observed rates of population decline. We used the range of rates reported in Curtis et al. (2014) as the minimum (63%) and maximum (73%) extent of decline and calculated an annual decline rate corresponding to each to set the bounds for MC sampling. This meant that our simulated population had to decline by the same total amount and over the same time period as in Curtis et al. (2014). In the backwards projections, the annual decline rate is defined as:

(3) $$\text{decline}=1-\frac{N_{t}}{N_{t-1}}$$

where population size in the previous year simply became population size in the current year divided by the corresponding rate of increase:

(4) $$N_{t-1}=\frac{N_{t}}{\left(1-\text{decline}\right)}$$

The rate of decline determines the difference between the number of animals in the population one year and abundance in the previous year (note that abundance in $N_{t-1}$ is always greater than in $N_{t}$ because the population is declining). For the population to be in decline, removals have to be greater than the difference between $N_{t-1}$ and $N_{t}$ because of annual population growth (*e^r^*):

(5) $$N_{t}=N_{t-1}e^{r}-\text{removals}$$

Given the definition of the annual decline rate in Equation 3, the annual removals necessary to cause a specific decline rate become:

(6) $${\text{removals}}_{t}=N_{t}\left(-1+e^{r}+\text{decline}\right).$$

To prevent overestimation of annual removals, we removed biologically implausible values of *r* by limiting its potential distribution between 0 and 2 times the deterministic estimate for the short and long lifespan scenarios, respectively. For each life history, the deterministic value for *r* was calculated directly from the median life history parameter values (Table 1). Because this is a density‐independent model, *r* did not change systematically with population size as would be expected under a compensatory model (Gedamke et al., 2007; Smith et al., 2008). Although this would have minimal effect on removal estimates when the population was small, removals may have been slightly overestimated when the population size was larger (i.e., when *r* would be expected to be lower).

To conceptualize how significant annual removals may have been to the population, we calculated a common data‐limited reference value, called Potential Biological Removals (PBR; Wade, 1998) for comparison. Originally developed for marine mammals, this reference point applies to species with uncertain population sizes yet the data necessary to estimate *r* and removals. The PBR represents an upper limit for annual mortality, expressed as a number of animals. Removals above this level are expected to lead to population decline. It is defined as:

(7) $$\text{PBR}=\frac{1}{2}rN_{\min}f.$$

Here, $N_{\min}$ is a conservative estimate of population size, typically approximated as the 20th percentile of a population size estimate, and *f* is a recovery factor that can take values between 0.1 and 1. Low values of *f* are recommended for threatened or endangered species (0.5 and 0.1, respectively) to account for any imprecision in the abundance estimate or imperfect knowledge of the mortality affecting the population (Dillingham & Fletcher, 2011; Lonergan, 2011). Canada considers the Northwest Atlantic population of white shark to be endangered (COSEWIC, 2006), so we have calculated the PBR using a recovery factor of 0.1.

The PBR depends on *N*_min_, which means that its value changes annually in our analyses as population size changes. The MC sampling in the life table analyses gave a distribution of *r* values that we used in the population projections to obtain a distribution of population sizes ( $N_{t}$ ) in a given year. We used the median value for *r* as well as the 20th percentile of the annual distribution of population sizes as $N_{\min}$ .

### 2.3 Population increase

The magnitude of recent population increase for Northwest Atlantic white shark is uncertain, but is corroborated by many types of data. From the hierarchical model developed by Curtis et al. (2014), both the annual posterior means and the estimated trend from locally weighted polynomial regression suggest that the population has approximately doubled from its minimum size in the 1980s. However, the authors considered the magnitude to be sufficiently uncertain that they limited their interpretation of the analysis to be indicative of a recent population increase. Projecting the simulation model forward allowed us to characterize the probability of achieving population growth in each of the two longevity scenarios. In addition, we were able to assess how effective conservation efforts may have been in reducing fishing mortality by approximating the level of removals that would allow such population increase.

As in the backwards projections, the simulation model projects the population forward from the assumed minimum size, representing postdecline abundance in 1990. Here, the effect of historical fisheries removals is incorporated into the calculation of survival by age (as in Equation 2) to determine *r* while future fisheries removals are incorporated as in Equation 5. To increase the biological realism of the projections, we incorporated annual environmental variability in *r* through adding autocorrelated deviates ( $w_{t}$ ). As in Hilborn (2001), these were calculated as:

(8) $$w_{t}=w_{t-1}d+w_{t}^{*}\sigma .$$

where

(9) $$w_{t}^{*}∼N\left(0,1\right)$$

The strength of autocorrelation ( d ) and the level of variability ( σ ) were both set at 0.03 in the projections. This low level of variability reflects the expectation that reproductive output is fairly constant for the Northwest Atlantic population of white shark, relative to the recruitment variability of other fish species (Kindsvater et al., 2018).

We summarized trends in the population trajectories using lognormal regression:

(10) $$\ln\left(N_{t}\right)=\alpha +\beta t,$$

where fitted slopes represent annual population growth rates. For the two longevity scenarios, we summarized the proportion of trajectories that had positive slopes (indicative of population increase), the proportion that increased by 10%, and the proportion that doubled in abundance. For each, we assumed that population increase took place over 30 years, beginning in 1990 and continuing to the present day. We sequentially decreased fishery removals relative to those which caused historic abundance declines to evaluate how effective national and international protective measures for white shark may have been. These results were also summarized relative to the proportion of trajectories that were positive, increased by 10%, and doubled, considering reductions in fishing pressure of 30%, 50%, 70% and 90% and 100%.

## 3 RESULTS

### 3.1 Capacity for growth

**FIGURE 1:**
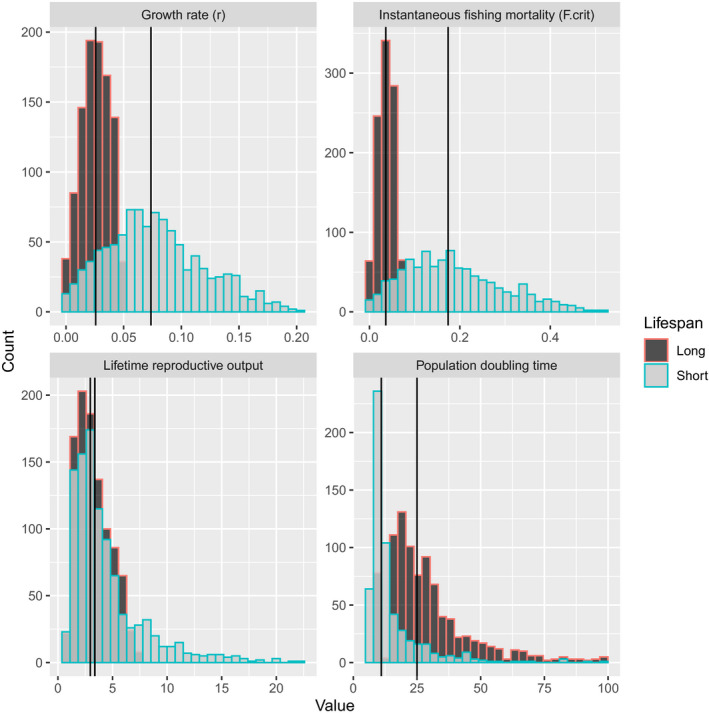
Distributions and median values (vertical lines) for demographic characteristics derived from the life history parameter values in Table 1, showing the difference between the short and long lifespan scenarios for white shark in the Northwest Atlantic

Greater longevity coupled with higher age at maturity in a white shark population dramatically reduces its annual rate of population increase. Estimates of *r* declined by 68% for the long lifespan scenario as compared to the short lifespan (Figure 1), changing from 0.074 (80th percentiles = 0.024, 0.139) to 0.026 (percentiles = 0.008, 0.042). Assuming no fishing mortality, the lower *r* from the long lifespan nearly triples the median number of years required for the population to double (c.f. 26 versus 9; Figure 1). Also, the amount of juvenile fishing mortality that would prevent population increase ( $F_{\text{crit}}$ ) declines from 0.174 (80th percentiles = 0.056, 0.342) to 0.036 (percentiles = 0.012, 0.060; Figure 1). However, the median lifetime reproductive output of females was only marginally higher for a population with a short lifespan (c.f. 3.37 versus 2.96; Figure 1). Given the life history values in Table 1, the simulations indicate that a female will produce approximately three adult female offspring throughout her life. The key life history differences between the scenarios are that females have higher survival at age and are reproductively active for an additional 10 years in the long lifespan scenario. It is interesting that these two factors combine to make lifetime reproductive rates similar between the two scenarios even though annual population growth rates (*r*) are so much lower in the long lifespan scenario.

In allowing for variability, there were several combinations of life history parameter values in both longevity scenarios that resulted in biological implausible estimates of *r* (e.g., *r* < 0). This was expected given the compounding nature of variability when uncertainty from multiple sources is combined. The parameter values associated with biologically implausible estimates for *r* were similar for the short and long lifespan parameterizations of the Euler–Lotka equation, and here we summarize the results for the long life history. It is important to remember that gestation time and reproductive periodicity were the same for both scenarios. As expected, the distributions of the life history parameters that gave plausible estimates for *r* centered on the median values used in the analyses. Negative estimates of *r* were associated with litter sizes on the lower boundary (2 females per litter), a reproductive periodicity on the upper boundary (3 years), and a high age at maturity (a median value of 34 years). Conversely, extremely high estimates of *r* were associated with litter sizes in the upper half of the assumed range (4–6 female offspring or 8–12 total offspring) combined with a gestation time of 2 years and a low median age at maturity (26 years). The distribution for maximum age centered on the deterministic value regardless of the plausibility of the estimate for *r*.

### 3.2 Removals affecting the population trajectory

**FIGURE 2:**
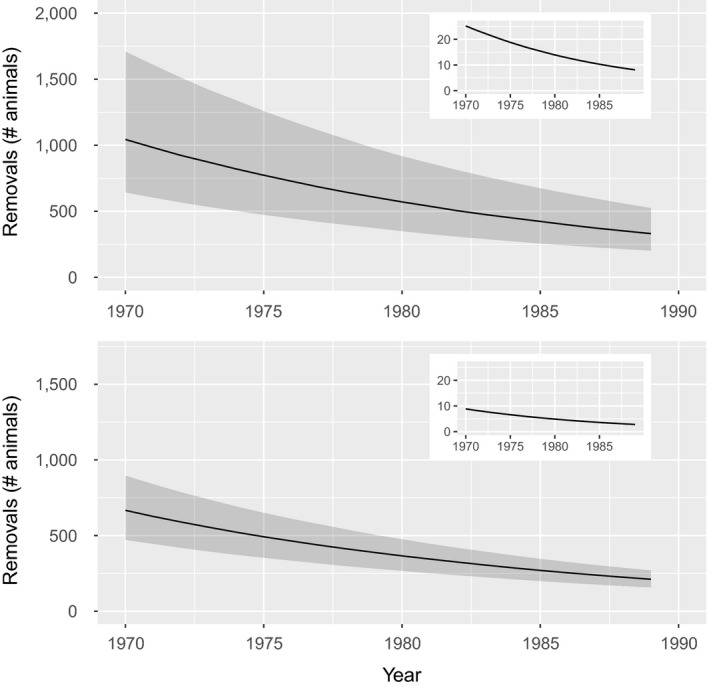
Predicted juvenile removals by year (main plots) relative to the Potential Biological Removals (PBR) reference point (inset plots) for the short life history (top panel) and long life history (bottom panel). Solid lines represent median values and the gray shading represents the 80th percentile. Note that the total decline rate as well as the years over which it occurred correspond to Curtis et al. (2014)

The total population decline estimate provided by in Curtis et al. (2014) results in an annual decline rate of 0.048–0.063 over 20 years (19‐time steps). If postdecline population size in the Northwest Atlantic had a median value of 2,500 animals (all life stages combined) in 1990, these rates imply a median predecline abundance of 7,834 (80th percentiles = 6,371, 9,587) in 1970. Fisheries removals of juvenile animals needed to be markedly higher in the short lifespan scenario to cause this abundance decline, reflecting the greater potential for annual population increase (Figure 2). Summing over the 20 years of decline, median removals in the short lifespan scenario (12,493 juveniles) were 1.6 times higher than in the long lifespan scenario (7,981 juveniles). Removals in any given year ranged from 1,044 fish (80th percentiles = 643, 1708) in 1970 to 330 fish (percentiles = 202, 525) in 1989 for the short lifespan scenario, and 667 fish (percentiles = 472, 895) to 212 fish (percentiles = 158, 269) for the long lifespan scenario. Similar to removals, the PBR reference point in any given year declined commensurate with the population (Figure 2) because the PBR represents a constant proportion of the population size. In all cases, the PBR was an extremely small number of animals, <1% of the predicted median annual population size. In order to cause the observed rates of population decline, removals would have needed to be 40–60 times higher than the annual PBR (Figure 2).

**FIGURE 3:**
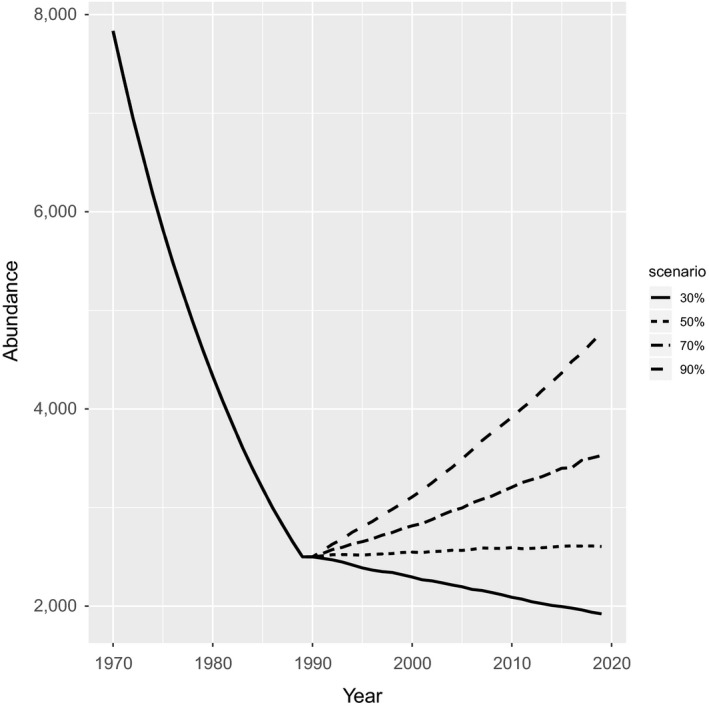
Historical population decline (1970–1989) and subsequent predicted population trajectory (1990–2019) under a 30%, 50%, 70%, or 90% reduction in fishing mortality for the long life history scenario

Recent research suggests that white shark in the Northwest Atlantic have been increasing in abundance since the 1990s (Curtis et al., 2014). In order for the majority of simulated white shark populations to do this, fishing mortality would have needed to be substantially lower than the levels that caused population declines. If fishing mortality rates declined by 30%, the median population trajectory under the long lifespan scenario would still be declining to the present day (Figure 3). In contrast, if fishing mortality was reduced by 90%, substantial population increase would have been possible (Figure 3), given that just under half of the projections doubled in abundance by 2015 (Table 2). To allow the majority of simulated trajectories to increase by 10% at the end of 30 years, removals needed to be reduced by approximately 30% for the short lifespan scenario and by 50% for the long lifespan (Table 2). For the majority of trajectories to have doubled in abundance, removals needed to be reduced by approximately 50% for the short lifespan scenario and by 100% for the long lifespan (Table 2).

**TABLE 2:** The proportion of simulated populations that increased by 10% or doubled in abundance over 30 years given various reductions in fishing mortality for the short and long lifespan scenarios of white shark in the Northwest Atlantic

Reduction in fishing mortality	Short lifespan		Long lifespan	
	Increased by 10%	Doubled	Increased by 10%	Doubled
30%	0.524	0.389	0.22	0.019
50%	0.702	0.549	0.452	0.075
70%	0.859	0.824	0.702	0.219
90%	0.925	0.845	0.872	0.461
100%	0.948	0.892	0.938	0.565

## 4 DISCUSSION

For white shark in the Northwest Atlantic, all scenarios examined suggest removals in the vicinity of 100s of juveniles per year could have led to observed historical population declines and thus may be cause for serious conservation concern if observed in future years. For the long lifespan, a 63%–73% decline over 20 years could have been caused by removals ranging from 667 to 211 juveniles per year. If white shark in the Northwest Atlantic are as long lived as recent research suggests (Natanson & Skomal, 2015), their population can withstand very little fisheries‐induced mortality. This is concerning given their widespread distribution throughout the Northwest Atlantic (Skomal et al., 2017) and their potential for interaction with multiple commercial fleets in both national and international waters (Queiroz et al., 2019). Incidental captures have occurred in many gear types: including trawl, gillnet, trap or weir, and longline fisheries (Curtis et al., 2014; DFO, 2017), and estimating total discards of white shark in the Northwest Atlantic has been flagged as a research priority (Curtis et al., 2018). Although incidental mortality from any one fleet might be expected to be rare and sporadic, there is currently no real method by which to track collective mortality from all fleets and fisheries affecting the population in the Northwest Atlantic. For comparison, collective mortality from the United States and Mexican fisheries (coastal plus high‐seas) for YOY and juvenile white sharks in the Northeast Pacific has been estimated at 208 individuals per year (Dewar et al., 2013). Removals in the 100s of animals are exceptionally low compared with levels that would cause severe decline in the majority of fish populations (Kindsvater et al., 2018). This speaks to the inherent vulnerability of white shark to fishing pressure and reaffirms the need for restrictive conservation measures to protect the population even under a situation of abundance increase (Dudley & Simpfendorfer, 2006).

Although population vital rates are uncertain and abundance of white shark in the Northwest Atlantic is unknown, it is likely that the magnitude of removals we report is realistic. In these simulations, the magnitude of removals is related to the population growth rate (*r*) as well as to the assumed population size, where removals are greater from larger populations and/or those characterized by a higher value for *r*. Both of the *r* estimates (short or long lifespan) fell within the 95% credible interval for the intrinsic rate of growth estimated for the Northeast Pacific population of white shark (0.02–0.091; Dillingham et al., 2016), with our long lifespan estimate falling closer to the mean (*c.f.* 0.027 and 0.05). The life history rates used to characterize the short lifespan were similar to those used in previous demographic analyses of rebound potential for white shark. As expected, our estimate for the short lifespan (*r* = 0.081) was higher than estimates for rebound potential (e.g., *r*_2_ *_M_* = 0.04, Smith et al., 1998) because the former is expected to represent the capacity of population growth at extremely low population size in the absence of density dependence, while the latter represents the intrinsic rate of population increase at MSY from a density‐dependent model (Au et al., 2015; Smith et al., 1998, 2008). Unlike in Dillingham et al. (2016), our primary goal was not to improve the precision of the estimate for *r*. Our consideration of the two lifespan scenarios was intended to demonstrate the specific impact that a re‐evaluation of age and growth can have on expected lifetime reproductive output and annual population growth rates, leading to a substantial reduction in the magnitude of annual removals that would cause population decline. Relative to population size, we based our simulated postdecline population size (i.e., population size in 1990) on abundance estimates from other areas, notably a new genetic estimate based on close‐kin‐mark‐recapture that suggests between 2,500 and 6,750 white shark inhabit Eastern Australian and New Zealand (Hillary et al., 2018), and a recent photo‐identification mark‐recapture estimate of >2,000 individuals off central California (Burgess et al., 2014). We recognize that these estimates represent specific aggregations rather than whole‐ocean populations of white shark. However, if postdecline population sizes were higher than assumed here, the magnitude of annual removals would scale proportionately and become similar to values predicted in earlier years from the backwards projections.

That our understanding of longevity can change so significantly has implications for research aimed at describing other population vital rates, most notably reproduction. As reviewed by Bruce (2008), exceptionally few pregnant female white shark have been reliably examined, resulting in little data on embryonic development rates or reproductive periodicity (Christiansen et al., 2014). Observed litter sizes range from 2 to 17 pups, with the expectation that the lower values represent situations of loss through spontaneous abortion. Estimates of ~10 pups (50:50 sex ratio) are thought to be the most representative (Bruce, 2008; Francis, 1996). Given this level of reproductive output, our results favor the interpretation that white shark have a reproductive cycle longer than 2 years. In our simulations, implausibly high estimates of *r* were associated with litter sizes that are expected to be representative (8–12 pups) coupled with a reproductive periodicity of 2 years. These results flag research on reproductive dynamics (e.g., maturation, senescence, reproductive periodicity, and the potential for resting between reproduction events) as critical given the possibility of increased longevity (Huveneers et al., 2018). Exploring such questions for white shark will require further development and validation of nonlethal sampling methodologies, such as hormonal analysis coupled with ultrasound imaging (Hammerschlag & Sulikowski, 2011).

It can be difficult to determine if apparent changes in abundance are primarily associated with differences in actual abundance or with shifts in the availability of the population to enumeration (Maunder & Punt, 2004; Maunder et al., 2006; Thorson, Fonner, Haltuch, Ono, & Winker, 2016). This is particularly problematic when using fishery‐dependent data (e.g., catch‐per‐unit‐effort) from a restricted portion of the animal's total range to index population trends. Our analyses suggest that the white shark population in the Northwest Atlantic is unlikely to have doubled in abundance over the last 30 years, given what we now know about life history. For the long lifespan scenario, conservation actions would have needed to be nearly 100% effective at eliminating mortality from fisheries (including any incidental or nontarget captures of juveniles) in order for the majority of projections to double. Even to allow increases of 10% over 30 years required fishing mortality on juveniles to be reduced by greater than 50%. Recent trend information was based on catch‐per‐unit‐effort data from the Northeast Fisheries Science Center pelagic shark longline survey as well as the directed shark longline fishery at‐sea observer program, corroborated by sightings records (Curtis et al., 2014). That the abundance index increased so rapidly from the 1990s suggests that climactic or environmental variation has affected the distribution of white shark in the Northwest Atlantic and thus encounter probabilities in the fishery‐independent data (e.g., Hobday & Evans, 2013), or that there have been changes in fleet behavior that increase susceptibility to capture (e.g., Tidd, Brouwer, & Pilling, 2017). The recent trend is very unlikely to be solely due to changes in abundance over time.

Even with uncertainty in the degree of abundance change, it is heartening that the conservation measures put in place to protect white shark have likely been effective in reducing fishing mortality in the Northwest Atlantic. These include: prohibited species designation in the US (1997), listing on Appendix II of the Convention on International Trade in Endangered Species of Wild Fauna and Flora (CITES; 2005) as well as on Appendix II and III of the Convention on the Conservation of Migratory Species (CMS; 2002), and listing on Schedule I of the Canadian Species at Risk Act (SARA; 2011) (Christiansen et al., 2014; Curtis et al., 2014; DFO, 2017; Hillary et al., 2018). However, there is still a very long way to go if our goal is to keep total removals below the PBR reference point on an annual basis. Even at predecline population sizes (corresponding to *N*_min_ = 6,889), the PBR reference point was 25 animals for the short lifespan scenario and 9 animals for the long lifespan scenario. At that time, it is unlikely that white shark in the Northwest Atlantic would have been considered endangered when calculating the PBR, so a higher recovery factor would have been assumed (0.5; Dillingham & Fletcher, 2008; Wade, 1998). Even using *f* = 0.5 gives a PBR equal to 126 or 44 animals, respectively. “Every fish counts” is likely the perspective with which individual resource managers should view white shark mortalities in the Northwest Atlantic.

## CONFLICT OF INTEREST

None declared.

## AUTHOR CONTRIBUTION

**Heather D. Bowlby:** Conceptualization (lead); formal analysis (lead); Methodology (equal); writing – original draft (lead); Writing – review and editing (lead). **A. Jamie F. Gibson:** Conceptualization (supporting); formal analysis (supporting); methodology (equal); writing – review and editing (supporting).

## Data Availability

There are no data that accompanies this manuscript. Simulation code is available through Dryad: https://doi.org/10.5061/dryad.vhhmgqnqk
